# *N*-(2-Pyridylmeth­yl)phthalimide

**DOI:** 10.1107/S160053680903846X

**Published:** 2009-09-30

**Authors:** Olga Garduño-Beltrán, Perla Román-Bravo, Felipe Medrano, Hugo Tlahuext

**Affiliations:** a Centro de Investigaciones Químicas, Universidad Autónoma del Estado de Morelos. Av. Universidad 1001 Col., Chamilpa, CP 62209, Cuernavaca Mor., Mexico

## Abstract

In the title compound, C_14_H_10_N_2_O_2_, the phtalimide and 2-pyridylmethyl units are almost perpendicular, with an inter­planar angle of 85.74 (2)°. In the crystal, mol­ecules are linked by weak C—H⋯O inter­actions, forming chains running along the *b* axis. The packing is further stabilized by offset π–π inter­actions between adjacent pyridine rings, with a centroid–centroid distance of 3.855 (2) Å.

## Related literature

For general backround to phthalimides, see: Ing & Manske (1926 ▶); Gibson & Bradshaw (1968 ▶); Ishii & Sakaguchi (2004 ▶). For their applications in photochemical synthesis and catalytic and chiral reactions, see: Yoon & Mariano (2001 ▶); Huang *et al.* (2006 ▶); Rodríguez *et al.* 2006 ▶. For their biological activity, see: Miyachi *et al.* (1997 ▶); Vázquez *et al.* (2005 ▶). For phthalimide derivatives, see: Vamecq *et al.* (2000 ▶). For analysis of hydrogen-bonding patterns, see: Hunter (1994 ▶); Desiraju (1991 ▶); Bernstein *et al.* (1995 ▶).
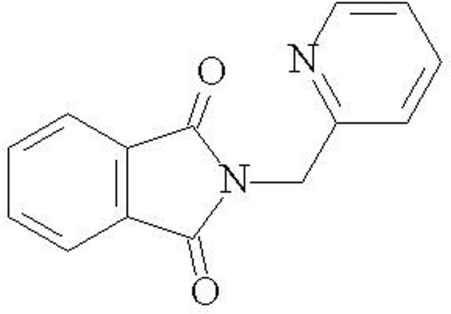


## Experimental

### 

#### Crystal data

C_14_H_10_N_2_O_2_
*M* *_r_* = 238.24
Monoclinic, 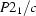

*a* = 11.7734 (18) Å
*b* = 14.239 (2) Å
*c* = 7.0698 (11) Å
β = 106.373 (3)°
*V* = 1137.1 (3) Å^3^
*Z* = 4
Mo *K*α radiation
μ = 0.10 mm^−1^
*T* = 293 K
0.45 × 0.28 × 0.19 mm

#### Data collection

Bruker SMART APEX CCD area-detector diffractometer
Absorption correction: multi-scan (*SADABS*; Sheldrick, 1996 ▶) *T*_min_ = 0.958, *T*_max_ = 0.982
7150 measured reflections
1994 independent reflections
1567 reflections with *I* > 2σ(*I*)
*R*_int_ = 0.028

#### Refinement

*R*[*F* ^2^ > 2σ(*F* ^2^)] = 0.063
*wR*(*F* ^2^) = 0.155
*S* = 1.20
1994 reflections
163 parameters
H-atom parameters constrained
Δρ_max_ = 0.18 e Å^−3^
Δρ_min_ = −0.29 e Å^−3^

### 

Data collection: *SMART* (Bruker, 2000 ▶); cell refinement: *SAINT-Plus*-NT (Bruker, 2001 ▶); data reduction: *SAINT-Plus*-NT; program(s) used to solve structure: *SHELXTL-NT* (Sheldrick, 2008 ▶); program(s) used to refine structure: *SHELXTL-NT*; molecular graphics: *SHELXTL-NT*; software used to prepare material for publication: *PLATON* (Spek, 2009 ▶) and *publCIF* (Westrip, 2009 ▶).

## Supplementary Material

Crystal structure: contains datablocks I, global. DOI: 10.1107/S160053680903846X/fl2266sup1.cif

Structure factors: contains datablocks I. DOI: 10.1107/S160053680903846X/fl2266Isup2.hkl

Additional supplementary materials:  crystallographic information; 3D view; checkCIF report

## Figures and Tables

**Table 1:** Hydrogen-bond geometry (Å, °)

*D*—H⋯*A*	*D*—H	H⋯*A*	*D*⋯*A*	*D*—H⋯*A*
C3—H3⋯O2^i^	0.93	2.53	3.452 (3)	171
C6—H6⋯O1^ii^	0.93	2.53	3.452 (3)	171
C14—H14⋯O1^iii^	0.93	2.65	3.373 (3)	135
C11—H11⋯O2^iv^	0.93	2.57	3.401 (3)	148

Symmetry codes: (i) 
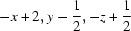
; (ii) 
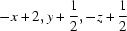
; (iii) 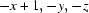; (iv) 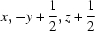.

**Fig. 1.**
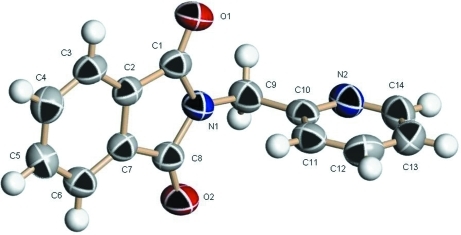
The molecular structure of (I), showing the atom-labelling scheme. Displacement ellipsoids are drawn at the 50% probability level and H atoms are shown as small spheres of arbitrary radius.

**Fig. 2.**
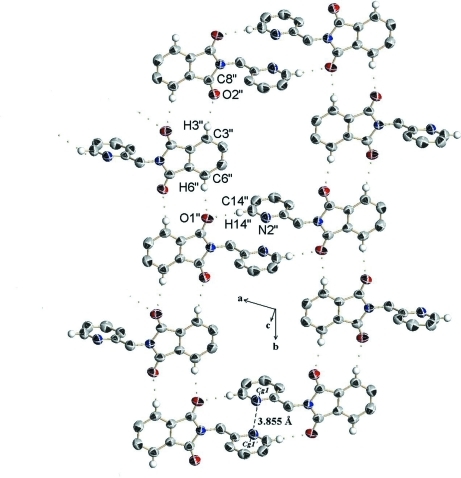
A view of the hydrogen bonds (dotted lines) in (I), showing the C3—H3···O2, C6—H6···O1, C14—H14···O1 interactions and the R22(10), R22(16), R64(30) motifs. Dashed line indicates the vector between pyridyl centroids (Cg1, Cg1').

## supplementary crystallographic information

### Comment

Phthalimides are indispensable in protection and deprotection of primary amines (Ing & Manske, 1926; Gibson & Bradshaw, 1968; Ishii & Sakaguchi, 2004). Phthalimide derivatives are useful in photochemical synthesis (Yoon & Mariano, 2001) and catalytic reactions (Huang *et al.*, 2006; Rodríguez *et al.*, 2006). Some of the phthalimide derivatives have applications as drugs (Vamecq *et al.*, 2000). Thus, they have been used as novel biological modifiers for tumor necrosis (Miyachi *et al.*, 1997). Their fluorescence properties  are highly environment sensitive (Vázquez *et al.*, 2005) and find application as biological probes. In our ongoing research on phthalimides as intermediates in supramolecular host design, we have synthesized the title compound, (I).

In  (I), all bond lengths and angles show normal values. The phtalimide and 2-pyridylmethyl moieties are almost pependicular with an interplanar angle of 85.74° (Fig.1).

In the crystal, molecules are linked by weak C—H···O interactions (Table 1) (Desiraju, 1991; Hunter, 1994), forming chains running along the *b* axis.

In the hydrogen-bonding pattern, two graph sets (Bernstein *et al.*, 1995) can be distinguished: *R*_2_^2^(10), involving atoms (···H6/C6—C8/O2···H3/C3—C1/O1)and *R*_2_^2^(16), involving atoms (···H14/C14/N2/C10/C9/N1/C1/O1···)_2_. Both patterns *R*_2_^2^(10) and *R*_2_^2^(16) are linked through weak C—H···O···H—C three center interactions, generating a motif belonging to the unitary graph set *R*_6_^4^(30) (Fig. 2).

The packing is further stabilized by aromatic π-π interactions, with distances between the centroids of the pyridine rings [*Cg*1, *Cg*1'^i^ (symmetry code: (i) 1 - *x*, -*y*, -*z*)] of 3.855 Å (Fig. 2).

### Experimental

A solution of 2-aminomethyl-pyridine (1 g, 9.25 mmol) in dimethylformamide(DMF) (5 ml) was added dropwise to (1.36 g, 9.18 mmol) of phthalic anhydride dissolved in 10 ml of DMF and refluxed for 6 h. The resulting solution was concentrated under reduced pressure to a viscous yellow liquid. Addition of water (25 ml) gave a colorless solid which was recovered by filtration and dried under vacuum. The product was recrystallized from ethanol to give suitable crystals for X-ray diffraction analysis (m.p. 399 K)

### Refinement

Non-hydrogen atoms were refined anisotropically. Aromatic and methylene H atoms were positioned geometrically and constrained using the riding-model approximation [*C*—H_aryl_ = 0.93 Å, *U*_iso_(H_aryl_) = 1.2 *U*_eq_(*C*_aryl_); *C*—H_methylene_ = 0.97 Å, *U*_iso_(H_methylene_) = 1.2 *U*_eq_(C_methylene_)], but the coordinates were refined freely.

### Crystal data

**Table:**

C_14_H_10_N_2_O_2_	*F*(000) = 496
*M**_r_* = 238.24	*D*_x_ = 1.392 Mg m^−^^3^
Monoclinic, *P*2_1_/*c*	Melting point: 399 K
Hall symbol: -P 2ybc	Mo *K*α radiation, λ = 0.71073 Å
*a* = 11.7734 (18) Å	Cell parameters from 2665 reflections
*b* = 14.239 (2) Å	θ = 2.3–26.8°
*c* = 7.0698 (11) Å	µ = 0.10 mm^−^^1^
β = 106.373 (3)°	*T* = 293 K
*V* = 1137.1 (3) Å^3^	Prism, colourless
*Z* = 4	0.45 × 0.28 × 0.19 mm

### Data collection

**Table:**

Bruker SMART APEX CCD area-detector diffractometer	1994 independent reflections
Radiation source: fine-focus sealed tube	1567 reflections with *I* > 2σ(*I*)
graphite	*R*_int_ = 0.028
Detector resolution: 8.3 pixels mm^-1^	θ_max_ = 25.0°, θ_min_ = 1.8°
φ and ω scans	*h* = −13→13
Absorption correction: multi-scan (*SADABS*; Sheldrick, 1996)	*k* = −16→16
*T*_min_ = 0.958, *T*_max_ = 0.982	*l* = −8→8
7150 measured reflections	

### Refinement

**Table:**

Refinement on *F*^2^	Primary atom site location: structure-invariant direct methods
Least-squares matrix: full	Secondary atom site location: difference Fourier map
*R*[*F*^2^ > 2σ(*F*^2^)] = 0.063	Hydrogen site location: inferred from neighbouring sites
*wR*(*F*^2^) = 0.155	H-atom parameters constrained
*S* = 1.20	*w* = 1/[σ^2^(*F*_o_^2^) + (0.0705*P*)^2^ + 0.1417*P*] where *P* = (*F*_o_^2^ + 2*F*_c_^2^)/3
1994 reflections	(Δ/σ)_max_ < 0.001
163 parameters	Δρ_max_ = 0.18 e Å^−^^3^
0 restraints	Δρ_min_ = −0.29 e Å^−^^3^

### Special details

**Table:**

Geometry. All e.s.d.'s (except the e.s.d. in the dihedral angle between two l.s. planes) are estimated using the full covariance matrix. The cell e.s.d.'s are taken into account individually in the estimation of e.s.d.'s in distances, angles and torsion angles; correlations between e.s.d.'s in cell parameters are only used when they are defined by crystal symmetry. An approximate (isotropic) treatment of cell e.s.d.'s is used for estimating e.s.d.'s involving l.s. planes.
Refinement. Refinement of *F*^2^ against ALL reflections. The weighted *R*-factor *wR* and goodness of fit *S* are based on *F*^2^, conventional *R*-factors *R* are based on *F*, with *F* set to zero for negative *F*^2^. The threshold expression of *F*^2^ > σ(*F*^2^) is used only for calculating *R*-factors(gt) *etc*. and is not relevant to the choice of reflections for refinement. *R*-factors based on *F*^2^ are statistically about twice as large as those based on *F*, and *R*- factors based on ALL data will be even larger.

### Fractional atomic coordinates and isotropic or equivalent isotropic displacement parameters (Å^2^)

**Table:**

	*x*	*y*	*z*	*U*_iso_*/*U*_eq_	
C1	0.8978 (2)	0.03798 (15)	0.1337 (3)	0.0493 (6)	
C2	1.01324 (19)	0.07017 (14)	0.2618 (3)	0.0456 (5)	
C3	1.1103 (2)	0.01983 (16)	0.3657 (3)	0.0533 (6)	
H3	1.1100	−0.0455	0.3665	0.064*	
C4	1.2086 (2)	0.07039 (18)	0.4690 (3)	0.0613 (7)	
H4	1.2760	0.0384	0.5399	0.074*	
C5	1.2088 (2)	0.16764 (17)	0.4691 (3)	0.0612 (6)	
H5	1.2761	0.1997	0.5405	0.073*	
C6	1.1106 (2)	0.21791 (16)	0.3648 (3)	0.0557 (6)	
H6	1.1105	0.2832	0.3646	0.067*	
C7	1.01340 (18)	0.16754 (14)	0.2616 (3)	0.0466 (5)	
C8	0.8971 (2)	0.19976 (15)	0.1347 (3)	0.0515 (6)	
C9	0.7148 (2)	0.11883 (15)	−0.0729 (3)	0.0576 (6)	
H9A	0.7048	0.0621	−0.1517	0.069*	
H9B	0.7075	0.1718	−0.1617	0.069*	
C10	0.61635 (19)	0.12393 (13)	0.0243 (3)	0.0484 (6)	
C11	0.6362 (2)	0.13358 (14)	0.2231 (3)	0.0573 (6)	
H11	0.7128	0.1380	0.3066	0.069*	
C12	0.5388 (3)	0.13668 (17)	0.2973 (4)	0.0705 (7)	
H12	0.5489	0.1437	0.4319	0.085*	
C13	0.4280 (2)	0.12929 (16)	0.1695 (5)	0.0708 (7)	
H13	0.3614	0.1303	0.2156	0.085*	
C14	0.4168 (2)	0.12042 (16)	−0.0258 (4)	0.0672 (7)	
H14	0.3410	0.1159	−0.1118	0.081*	
N1	0.83334 (16)	0.11904 (11)	0.0626 (3)	0.0530 (5)	
N2	0.50806 (18)	0.11779 (12)	−0.1016 (3)	0.0610 (6)	
O1	0.86171 (15)	−0.04110 (11)	0.0924 (2)	0.0652 (5)	
O2	0.85977 (15)	0.27886 (11)	0.0976 (2)	0.0681 (5)	

### Atomic displacement parameters (Å^2^)

**Table:**

	*U*^11^	*U*^22^	*U*^33^	*U*^12^	*U*^13^	*U*^23^
C1	0.0604 (15)	0.0411 (13)	0.0521 (13)	−0.0013 (10)	0.0249 (11)	0.0003 (10)
C2	0.0532 (13)	0.0419 (12)	0.0483 (12)	0.0033 (10)	0.0251 (10)	0.0040 (9)
C3	0.0655 (16)	0.0427 (12)	0.0571 (13)	0.0090 (11)	0.0260 (12)	0.0072 (10)
C4	0.0564 (15)	0.0678 (17)	0.0592 (14)	0.0082 (12)	0.0154 (12)	0.0116 (12)
C5	0.0554 (15)	0.0658 (17)	0.0603 (14)	−0.0079 (12)	0.0126 (12)	0.0027 (12)
C6	0.0610 (15)	0.0447 (13)	0.0630 (14)	−0.0036 (11)	0.0201 (12)	0.0014 (11)
C7	0.0528 (13)	0.0407 (12)	0.0507 (12)	0.0014 (9)	0.0217 (10)	0.0034 (9)
C8	0.0566 (14)	0.0404 (13)	0.0608 (14)	0.0018 (10)	0.0218 (11)	0.0040 (10)
C9	0.0572 (14)	0.0559 (15)	0.0555 (13)	−0.0027 (11)	0.0088 (11)	0.0002 (10)
C10	0.0528 (13)	0.0335 (12)	0.0540 (12)	0.0012 (9)	0.0069 (10)	0.0016 (9)
C11	0.0575 (14)	0.0506 (14)	0.0571 (13)	−0.0010 (11)	0.0052 (11)	−0.0051 (10)
C12	0.088 (2)	0.0634 (17)	0.0610 (15)	0.0016 (14)	0.0220 (15)	−0.0102 (12)
C13	0.0612 (16)	0.0625 (17)	0.091 (2)	0.0054 (12)	0.0250 (15)	−0.0024 (14)
C14	0.0513 (15)	0.0626 (17)	0.0794 (18)	−0.0002 (11)	0.0050 (13)	0.0013 (13)
N1	0.0516 (11)	0.0409 (11)	0.0647 (12)	−0.0015 (8)	0.0135 (9)	0.0013 (8)
N2	0.0555 (12)	0.0563 (13)	0.0627 (12)	−0.0001 (9)	0.0029 (10)	0.0025 (9)
O1	0.0795 (12)	0.0428 (10)	0.0732 (11)	−0.0070 (8)	0.0214 (9)	−0.0059 (8)
O2	0.0689 (11)	0.0409 (10)	0.0873 (12)	0.0063 (8)	0.0100 (9)	0.0071 (8)

### Geometric parameters (Å, °)

**Table:**

C1—O1	1.210 (3)	C8—N1	1.389 (3)
C1—N1	1.395 (3)	C9—N1	1.453 (3)
C1—C2	1.477 (3)	C9—C10	1.506 (3)
C2—C3	1.374 (3)	C9—H9A	0.9700
C2—C7	1.386 (3)	C9—H9B	0.9700
C3—C4	1.383 (3)	C10—N2	1.336 (3)
C3—H3	0.9300	C10—C11	1.366 (3)
C4—C5	1.385 (4)	C11—C12	1.391 (3)
C4—H4	0.9300	C11—H11	0.9300
C5—C6	1.382 (3)	C12—C13	1.365 (4)
C5—H5	0.9300	C12—H12	0.9300
C6—C7	1.373 (3)	C13—C14	1.356 (4)
C6—H6	0.9300	C13—H13	0.9300
C7—C8	1.483 (3)	C14—N2	1.329 (3)
C8—O2	1.210 (2)	C14—H14	0.9300
			
O1—C1—N1	124.4 (2)	N1—C9—H9A	108.6
O1—C1—C2	129.5 (2)	C10—C9—H9A	108.6
N1—C1—C2	106.10 (18)	N1—C9—H9B	108.6
C3—C2—C7	121.4 (2)	C10—C9—H9B	108.6
C3—C2—C1	130.5 (2)	H9A—C9—H9B	107.5
C7—C2—C1	108.13 (18)	N2—C10—C11	123.1 (2)
C2—C3—C4	117.2 (2)	N2—C10—C9	113.95 (19)
C2—C3—H3	121.4	C11—C10—C9	122.9 (2)
C4—C3—H3	121.4	C10—C11—C12	118.1 (2)
C3—C4—C5	121.5 (2)	C10—C11—H11	120.9
C3—C4—H4	119.3	C12—C11—H11	120.9
C5—C4—H4	119.3	C13—C12—C11	118.9 (3)
C6—C5—C4	121.1 (2)	C13—C12—H12	120.5
C6—C5—H5	119.4	C11—C12—H12	120.5
C4—C5—H5	119.4	C14—C13—C12	118.8 (2)
C7—C6—C5	117.3 (2)	C14—C13—H13	120.6
C7—C6—H6	121.3	C12—C13—H13	120.6
C5—C6—H6	121.3	N2—C14—C13	123.8 (2)
C6—C7—C2	121.5 (2)	N2—C14—H14	118.1
C6—C7—C8	130.5 (2)	C13—C14—H14	118.1
C2—C7—C8	107.97 (19)	C8—N1—C1	111.69 (19)
O2—C8—N1	124.4 (2)	C8—N1—C9	124.25 (18)
O2—C8—C7	129.5 (2)	C1—N1—C9	124.06 (18)
N1—C8—C7	106.11 (18)	C14—N2—C10	117.2 (2)
N1—C9—C10	114.79 (19)		
			
O1—C1—C2—C3	0.9 (4)	N1—C9—C10—C11	3.7 (3)
N1—C1—C2—C3	−178.73 (19)	N2—C10—C11—C12	0.3 (3)
O1—C1—C2—C7	179.4 (2)	C9—C10—C11—C12	−179.42 (19)
N1—C1—C2—C7	−0.2 (2)	C10—C11—C12—C13	0.5 (3)
C7—C2—C3—C4	−0.5 (3)	C11—C12—C13—C14	−0.9 (4)
C1—C2—C3—C4	177.91 (19)	C12—C13—C14—N2	0.5 (4)
C2—C3—C4—C5	0.5 (3)	O2—C8—N1—C1	−178.6 (2)
C3—C4—C5—C6	−0.3 (3)	C7—C8—N1—C1	0.7 (2)
C4—C5—C6—C7	0.0 (3)	O2—C8—N1—C9	2.3 (3)
C5—C6—C7—C2	0.1 (3)	C7—C8—N1—C9	−178.38 (18)
C5—C6—C7—C8	−178.8 (2)	O1—C1—N1—C8	−180.0 (2)
C3—C2—C7—C6	0.2 (3)	C2—C1—N1—C8	−0.3 (2)
C1—C2—C7—C6	−178.53 (18)	O1—C1—N1—C9	−0.9 (3)
C3—C2—C7—C8	179.31 (18)	C2—C1—N1—C9	178.74 (18)
C1—C2—C7—C8	0.6 (2)	C10—C9—N1—C8	−87.1 (2)
C6—C7—C8—O2	−2.5 (4)	C10—C9—N1—C1	93.9 (2)
C2—C7—C8—O2	178.4 (2)	C13—C14—N2—C10	0.3 (3)
C6—C7—C8—N1	178.2 (2)	C11—C10—N2—C14	−0.7 (3)
C2—C7—C8—N1	−0.8 (2)	C9—C10—N2—C14	179.06 (18)
N1—C9—C10—N2	−176.06 (17)		

### Hydrogen-bond geometry (Å, °)

**Table:**

*D*—H···*A*	*D*—H	H···*A*	*D*···*A*	*D*—H···*A*
C3—H3···O2^i^	0.93	2.53	3.452 (3)	171
C6—H6···O1^ii^	0.93	2.53	3.452 (3)	171
C14—H14···O1^iii^	0.93	2.65	3.373 (3)	135
C11—H11···O2^iv^	0.93	2.57	3.401 (3)	148

Symmetry codes: (i) −*x*+2, *y*−1/2, −*z*+1/2; (ii) −*x*+2, *y*+1/2, −*z*+1/2; (iii) −*x*+1, −*y*, −*z*; (iv) *x*, −*y*+1/2, *z*+1/2.
